# Trajectories of Exposure to Neighborhood Deprivation and the Odds of Experiencing Intimate Partner Violence Among Women: Are There Sensitive Periods for Exposure?

**DOI:** 10.1177/0886260520959626

**Published:** 2020-09-22

**Authors:** Alexa R. Yakubovich, Jon Heron, Christine Barter, David K. Humphreys

**Affiliations:** 1 University of Oxford, Oxford, United Kingdom; 2 Li Ka Shing Knowledge Institute, St. Michael’s Hospital, Toronto, Ontario, Canada; 3 University of Bristol, Bristol, United Kingdom; 4 University of Central Lancashire, Preston, Lancashire, United Kingdom

**Keywords:** intimate partner violence, Neighborhoods, Longitudinal studies, Women, United Kingdom

## Abstract

Neighborhood disadvantage is commonly hypothesized to be positively associated with intimate partner violence (IPV) against women. However, longitudinal investigation of this association has been limited, with no studies on whether the timing of exposure matters. We used data from 2,115 women in the UK-based Avon Longitudinal Study of Parents and Children. Exposure to neighborhood-level deprivation was measured at 10-time points from baseline (gestation) until age 18. Family-level socioeconomic characteristics were measured at baseline. At age 21, participants self-reported whether they had experienced any IPV since age 18. We used a three-step bias-adjusted longitudinal latent class analysis to investigate how different patterns of neighborhood deprivation exposure were associated with the odds of experiencing IPV. A total of 32% of women experienced any IPV between ages 18 and 21. Women who consistently lived in deprived neighborhoods (chronic high deprivation) or spent their early childhoods in more deprived neighborhoods and later moved to less deprived neighborhoods (decreasing deprivation) had higher odds of experiencing IPV compared to those who consistently lived in non-deprived neighborhoods. The odds of experiencing IPV did not consistently differ between women who lived in non-deprived neighborhoods during early childhood and later moved to deprived neighborhoods (increasing deprivation) and those stably in non-deprived neighborhoods. Living in more deprived neighborhoods during early childhood, regardless of later exposure, was associated with higher odds of experiencing later IPV. This is congruent with prior research demonstrating the persistent effects of early neighborhood disadvantage on health and well-being. Replication, and underlying mechanisms, should be assessed across contexts.

## Introduction

Physical, psychological, or sexual violence committed by a current or former partner is one of the most common forms of violence against women, with severe consequences for health including death, injury, and mental health disorders (Campbell, 2002; Garcia-Moreno et al., 2013). Many multilevel theories on the causes of intimate partner violence (IPV) have hypothesized that neighborhood disadvantage increases the risk of experiencing or perpetrating this violence (Beyer et al., 2015; Vanderende et al., 2012; Voith, 2017). Although the cross-sectional literature has largely shown a positive association (Capaldi et al., 2012), longitudinal studies are limited, typically investigating only point-in-time or concurrent neighborhood exposures, and have shown mixed results (Benson et al., 2003; DeMaris et al., 2003; Fox et al., 2002; Giordano et al., 2016; Gomez, 2011; Jain et al., 2010; Leddy et al., 2018; Thulin et al., 2020; van Wyk et al., 2003; Yakubovich et al., 2018). Interrogating the longitudinal relationship between neighborhood disadvantage and IPV against women is critical to informing the design of structural interventions that can have wider population impacts in preventing IPV and its health consequences compared to targeted or individual-level interventions alone (Cerda et al., 2014; Cerda et al., 2015).

Social disorganization theory (Shaw & McKay, 1942)—or its extension, collective efficacy theory (Sampson et al., 1997)—is often used to hypothesize a positive relationship between neighborhood disadvantage and IPV (Beyer et al., 2015; Browning, 2002; Vanderende et al., 2012; Voith, 2017). Researchers postulate that neighborhoods with fewer socioeconomic resources and greater residential instability will be less able to establish the social ties and informal social control necessary to minimize violence and maximize intervention capacity. This is further extended in applications to IPV wherein stronger neighborhood social ties and support structures are hypothesized to guide women away from known violent partners, provide resources and supports for women to separate from violent partners, and create an overall inhibitory environment (e.g., where neighbors are, and perceived to be, aware of and willing to act on occurrences of IPV; Browning, 2002).

These hypotheses center on the influence of the current neighborhood environment in the likelihood of IPV occurring. Yet our recent longitudinal study in the UK found that greater cumulative exposure to neighborhood deprivation over the first 18 years of women’s lives increased their risk of experiencing IPV in early adulthood, accounting for family environment confounders over time (Yakubovich, Heron, Feder et al., 2020). This raises the potential importance of the developmental impacts of neighborhood disadvantage on IPV risk (i.e., beyond the current neighborhood environment), which has otherwise not been quantitatively explored in longitudinal studies (Yakubovich et al., 2018).

A growing body of research has demonstrated that exposure to deprived neighborhoods over childhood is negatively associated with later psychosocial well-being—for example, decreased educational attainment (Wodtke et al., 2011), increased odds of early parenthood (Wodtke, 2013), and worse mental health in adulthood (Wheaton & Clarke, 2003)—as well as multigenerational outcomes, including lower cognitive ability among children in the next generation (Sharkey & Elwert, 2011). These studies have suggested explanatory mechanisms, such as increased trauma and stress, reduced availability of social and economic resources, and socialization (e.g., lowered expectations of services, decreased self-efficacy). These pathways may also increase women’s vulnerability towards violent relationships and ability to safely separate from violent partners (including via poor mental health, a prospective risk factor for experiencing IPV; Yakubovich et al., 2018). Such proposed mechanisms are in line with the hypothesis of the intergenerational transmission of trauma, for which small, positive effects of experiencing family violence in childhood have been observed on future IPV perpetration and victimization, with the latter association showing stronger effect sizes for women versus men (and the opposite for perpetration; Smith-Marek et al., 2015). Social learning and the normalization of violence have been used to explain these findings, which may further apply to early exposure to neighborhood violence, often positively associated with neighborhood disadvantage (Herrenkohl et al., 2020; Sampson et al., 2002).

A recent qualitative study further illustrates the application of developmental neighborhood effects to IPV (Voith et al., 2019). Men in batterer intervention programs in the USA described processes in which their neighborhoods over childhood and adolescence were impactful in their development and behavior, which included establishing norms supportive of traditional gender roles, the social learning of violence, psychological trauma, and decreased interpersonal trust and safety. The researchers also interpreted the structural factors that shaped these neighborhood environments and processes, from mass incarceration to deindustrialization—highlighting that neighborhood disadvantage and its resulting outcomes are caused, and must be addressed, at the policy level. To the extent that women’s partners have similar neighborhood exposure histories, these findings also support a potential developmental influence of neighborhood disadvantage on women’s experiences of IPV.

The practice of analyzing average effects of cumulative exposure to neighborhood disadvantage, while illustrative of *duration* effects, does not account for potential differences based on the *timing* of exposure (Wheaton & Clarke, 2003). Therefore in the current study, we aimed to advance longitudinal understanding of developmental neighborhood effects on IPV by investigating whether different patterns in the timing of exposure to neighborhood deprivation over the first 18 years of life were differentially associated with the odds of experiencing IPV among women in early adulthood using longitudinal latent class analysis. Taking such a spatial-temporal, life-course approach is critical to understanding whether there are sensitive periods for exposure to neighborhood deprivation for IPV risk, with implications for theory and intervention development (Jivraj et al., 2019; Wheaton & Clarke, 2003).

## Method

We used data from the Avon Longitudinal Study of Parents and Children (ALSPAC), an ongoing prospective-longitudinal study. All pregnant women resident in one of three health districts in the former county Avon in the UK due between April 1, 1991, and December 31, 1992, were eligible to participate (Boyd et al., 2013; Fraser et al., 2013). Initially, 14,541 pregnant women (and their eventual babies) were enrolled. When the children of enrolled mothers were age 7, eligible mothers not enrolled were contacted, increasing the sample to 15,454 mothers (76% of all eligible) with 14,901 babies alive at age 1. These children comprise the ALSPAC birth cohort, 7,219 of which were girls (our target sample). The ALSPAC Ethics and Law Committee and Local Research Ethics Committees provided ethical approval. Participants provided informed consent following the recommendations of the ALSPAC Ethics and Law Committee at the time.

### Measures

At age 21, women responded to a validated 8-item scale on physical, psychological, and sexual IPV experiences before and/or after age 18 (Table 1, *α* = .95; Yakubovich et al., 2019). The measure was developed by a team of IPV researchers based on questionnaires used with young people (Barter et al., 2009; Barter et al., 2017) and a clinical sample in Bristol (Hester et al., 2015) and piloted for acceptability with the ALSPAC participant advisory group. Items were conceptually similar to those from existing IPV scales but with the benefit of not limiting measurement to conflicts or disagreements or overburdening participants with a large inventory of items (Yakubovich et al., 2019). Moreover, unlike most short-form IPV measures, the current measure captured physical, psychological, and sexual IPV. We analyzed any experience of IPV between ages 18 and 21 as a primary outcome, accounting for temporality and skew.

**Table 1.** IPV Items.

Item	How Often Altogether Have Any of Your Partners Ever Done Any of the Following to You and How Old Were You:	Type of IPV
1	Told you who you could see and where you could go and/or regularly checked what you were doing and where you were (by phone or text)?	Psychological
2	Made fun of you, called you hurtful names, shouted at you?	Psychological
3	Used physical force such as pushing, slapping, hitting, or holding you down?	Physical
4	Used more severe physical force such as punching, strangling, beating you up, hitting you with an object?	Physical
5	Pressured you into kissing/touching/something else?	Sexual/psychological
6	Physically forced you into kissing/touching/something else?	Sexual
7	Pressured you into having sexual intercourse?	Sexual/psychological
8	Physically forced you into having sexual intercourse?	Sexual

*Note*. For each victimization item, participants indicated the frequency of occurrence—where 0 = Never, 1 = Once, 2 = A few times, 3 = Often—and age of occurrence, where 1 = Under 18, 2 = Over 18, 3 = Both. The question prompt included the following definition for “partner:” “By partner we mean anyone you have ever been out with or had a relationship with, long-term or short-term (including one-night stands).”

We measured participants’ longitudinal exposure to neighborhood deprivation using the 2010 Indices of Multiple Deprivation (McLennan et al., 2011), which were available for 10-time points, every 1–3 years, from baseline (pregnancy) to age 18. The Indices measure deprivation across seven domains (income, employment, education, health, crime, housing, living environment) at the level of the lower-layer super output area (LSOA) in England (Table A1). LSOAs are census units containing approximately 1,500 residents or 650 households designed to approximate residential neighborhoods. Participants’ neighborhoods were determined from the ALSPAC address database, where addresses were regularly tracked to maintain communication. We had access to the quintile ranks of participants’ neighborhoods at each time point, which indicates the deprivation levels of each participant’s neighborhood relative to all other neighborhoods in England. To balance specificity and sensitivity, deprived neighborhoods were defined as those in the most deprived Quintiles 4 and 5 at each time point, as in prior ALSPAC studies (Yakubovich, Heron, Feder et al., 2020; Yakubovich, Heron, & Humphreys, 2020). This allowed for a more conservative test of exposure to more versus less severe neighborhood deprivation while maintaining response variation (the proportion of participants in Quintile 5 decreased to ~6% over time). Changes in relative neighborhood deprivation in the study area were minimal over the study period, especially in terms of neighborhoods transitioning from the most deprived quintiles (4–5) to the least (1–3; Bristol City Council, 2011).

To account for confounding, we controlled for socioeconomic and psychosocial characteristics of participants’ family environments, as reported by participants’ mothers at baseline. Using baseline data followed best practice on avoiding temporal overlap and over-adjustment (i.e., controlling for variables on the causal pathway, which may induce collider-stratification bias; Hernán et al., 2004; Nagin, 2005). We selected covariates that we hypothesized predicted baseline neighborhood selection and future experiences of IPV based on the literature and data availability in ALSPAC (Capaldi et al., 2012; Wheaton & Clarke, 2003; Yakubovich, Heron, Feder et al., 2020; Yakubovich et al., 2018). We accounted for: parental education (mother or her partner had higher than standard schooling qualifications: A-level or degree), parental social class (mother or partner were in partly or unskilled occupations based on the 1991 standard occupational classification), maternal marital status, maternal depressive symptoms (10-item Edinburgh Post-natal Depression Scale, *α* = .85; Cox et al., 1987)), recent residential mobility, maternal social support (10-item ALSPAC Social Network Index, *α* = .79), financial difficulties in affording basic needs (food, clothing, heating, accommodation, items for children), participant’s race/ethnicity (white versus ethno-racial minority, due to the high proportion of white participants in the sample), and number of children in the household.

### Analytic Strategy

Longitudinal latent class analysis is a person-centered modeling method that characterizes distinct patterns of within-participant change over time on a variable (here, exposure to neighborhood deprivation) to approximate regions of the unknown population distribution of change (Muthén & Muthén, 2017). It is well suited to repeated measures of binary variables and requires no distributional or time-related assumptions. We used the modal maximum likelihood three-step approach (Vermunt, 2010), which accounts for measurement error in the latent classes without altering the measurement model itself (Heron et al., 2015). First, we used Mplus to conduct an unconditional longitudinal latent class analysis: We estimated four classes of longitudinal exposure to neighborhood deprivation based on prior trajectory analyses of these data (Morris et al., 2018; Yakubovich, Heron, & Humphreys, 2020). Second, we estimated the association between the different patterns of neighborhood deprivation exposure and the odds of experiencing IPV, accounting for family environment confounders and the classification probabilities from Step 1. This allowed us to consider whether living in more versus less deprived neighborhoods over different developmental periods (i.e., early childhood, school-age, adolescence) was associated with differential odds of experiencing IPV in early adulthood. All models used maximum likelihood estimation, which is unbiased as long as data are missing at random (i.e., the likelihood of being missing is related to the observed data but not the missing values themselves). We further varied the extent of missing data excluded to determine model robustness balanced against classification certainty.

## Results

Our available sample was 6,442 women in ALSPAC who had at least one-time point of data on their exposure to neighborhood deprivation. Table 2 shows that most participants were white (94%) or had mothers who were married at baseline (76%), had (or their partners had) higher than standard school qualifications (56%), were in (or their partners were in) skilled occupations (76%), or had not recently moved house (88%). At baseline, participants’ mothers had relatively low depressive symptom scores (clinical cut-off is 13) and strong social networks on average. Participants’ families had a mean financial difficulties score of 2.87 (*SD* = 3.51), with 64% experiencing any financial difficulty in meeting basic needs. On average, there was one other child (*SD* = 0.94) in participants’ households.

**Table 2.** Sample Characteristics at Baseline (*N* = 6,442).

Variable	*N* respondents	*N* (%)	*M*(*SD*)
High parental education: Higher than standard schooling qualifications	5,655	3,142 (55.56)	–
Low parental social class: In partly or unskilled occupations	4,712	1,108 (23.51)	–
Mother married	5,908	4,518 (76.47)	–
Maternal depressive symptoms score, 0–30	5,448	–	6.89 (4.76)
Recently moved house	5,476	653 (11.92)	-
Maternal social network index, 0–30	5,615	–	22.36 (3.89)
Financial difficulties score, 0–15	5,826	–	2.87 (3.51)
White	5,979	5,678 (94.07)	–
Number of other children in the household	5,826	–	0.82 (0.94)

**Figure 1.**
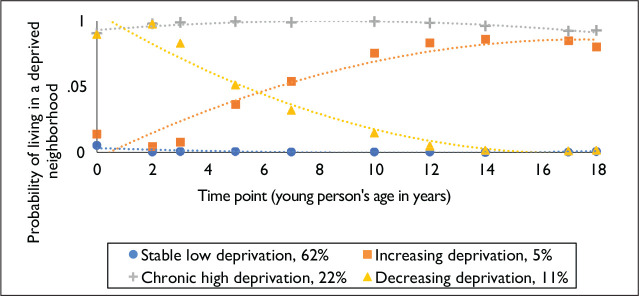
Longitudinal patterns of exposure to neighborhood deprivation (*N* = 6,442).

*Note*. Proportions for each trajectory group are based on the estimated model; dots represent observed means and dotted lines represent estimated trajectories. Entropy = .86. Stable low deprivation represents participants who consistently lived in non-deprived neighborhoods; chronic high deprivation represents participants who consistently lived in deprived neighborhoods; increasing deprivation shows participants who began living in less deprived neighborhoods and then moved to more deprived neighborhoods over the study period; decreasing deprivation shows the opposite pattern, where participants moved from more to less deprived neighborhoods over time.

Figure 1 shows the estimated longitudinal patterns of exposure to neighborhood deprivation experienced by women in the sample from in utero to age 18. Most participants consistently lived in non-deprived neighborhoods over the study period (stable low deprivation exposure, 62%). The next largest proportion of participants consistently lived in deprived neighborhoods (chronic high deprivation exposure, 22%). The remainder of participants lived in more deprived neighborhoods in childhood and moved to less deprived neighborhoods by adolescence (most by around age 10; decreasing deprivation exposure, 11%), or moved from non-deprived to deprived neighborhoods over this same period (increasing deprivation exposure, 5%). Classification certainty was reasonably strong regardless of whether we considered participants with at least one-time point of neighborhood deprivation data (as in Figure 1, entropy = .86) versus at least 50% of all available time points (as in Figure A1: entropy = .96). As demonstrated by Figure A1, the four-class solution was robust to varying amounts of missing data, with a slightly higher proportion of participants belonging to the normative class (stable low deprivation) when less versus more missing data were included (67% versus 62% of participants, respectively).

At age 21, *n* = 2,115 participants who had at least one-time point of neighborhood data reported on their experiences of IPV. Of these, 32% (*n* = 678) had experienced any IPV between ages 18 and 21. Table 3 summarizes the associations between the different longitudinal patterns of exposure to neighborhood deprivation until age 18 and the odds of experiencing IPV in early adulthood, accounting for baseline socioeconomic and psychosocial covariates and misclassification error. Compared to women who consistently lived in non-deprived neighborhoods throughout their childhoods (stable low deprivation), women who consistently lived in deprived neighborhoods (chronic high deprivation) had 53% higher odds of experiencing IPV in early adulthood (95% CI 1%, 132%). Likewise, women who spent their early childhoods in more deprived neighborhoods and then moved into less deprived neighborhoods (decreasing deprivation) had 66% higher odds (95% CI 8%, 156%) of experiencing IPV than those who consistently lived in non-deprived neighborhoods. In contrast, women who lived in less deprived neighborhoods during early childhood and later moved to more deprived neighborhoods (increasing deprivation) had higher odds (32%) of experiencing IPV compared to those stably living in non-deprived neighborhoods but the estimate was imprecise (95% CI –27%, 137%). These results were robust to including more versus less missing data (Tables A1–A3).

**Table 3.** Adjusted Association Between Latent Class of Neighborhood Deprivation Exposure Over First 18 Years of Life and the Odds of Experiencing IPV Between Ages 18 and 21 (*N* = 3,703).

Trajectory Group	Odds Ratio	95% CI
Stable low deprivation	Referent	
Increasing deprivation	1.32	.73–2.37
Chronic high deprivation	1.53	1.01–2.32
Decreasing deprivation	1.66	1.08–2.56

*Note*. *N* = 3,703 participants with at least 1-time point of neighborhood deprivation data over the study period and all baseline covariates. Model adjusts for all baseline covariates and misclassification bias (refer to method). Entropy = .79.

### Discussion

Women experienced distinct trajectories of neighborhood deprivation exposure throughout the first 18 years of their lives. A total of 72% lived stably in non-deprived neighborhoods (stable low deprivation); 22% lived consistently in deprived neighborhoods (chronic high deprivation); 11% lived in more deprived neighborhoods during early childhood, moving to less deprived neighborhoods by adolescence (decreasing deprivation); and 5% lived in less deprived neighborhoods in early childhood, moving to more deprived neighborhoods by adolescence (increasing deprivation). These longitudinal patterns of exposure were associated with differential odds of experiencing IPV in early adulthood. Accounting for family-level confounders, women who lived in deprived neighborhoods during their early childhoods, regardless of their later exposure (chronic high deprivation or decreasing deprivation), had 53%–66% higher odds of experiencing IPV as young adults than those who always lived in non-deprived neighborhoods. In contrast, women who moved from non-deprived to increasingly deprived neighborhoods did not consistently differ in their odds of experiencing IPV from those stably in non-deprived neighborhoods.

Existing longitudinal studies of neighborhood deprivation and IPV experiences among women have measured neighborhood deprivation exposure at most two times (Benson et al., 2003; Giordano et al., 2016; Gomez, 2011; Jain et al., 2010; Leddy et al., 2018; Yakubovich et al., 2018) or cumulatively (Yakubovich, Heron, Feder et al., 2020), precluding investigation of potential timing effects. The current study suggests that early childhood, as compared to adolescence, is a sensitive period for exposure in the association between neighborhood deprivation and IPV in early adulthood among women. This is congruent with prior research, which has demonstrated persistent effects of early neighborhood deprivation exposure on certain health and well-being outcomes (Sharkey & Elwert, 2011; Sharkey & Faber, 2014; Wheaton & Clarke, 2003). For instance, one of the first investigations of spatial-temporal neighborhood effects showed that childhood neighborhood disadvantage was associated with worse mental health (including internalizing problems, such as low self-worth, anxiety, feeling unloved) in early adulthood, over and above participants’ current neighborhood contexts (Wheaton & Clarke, 2003). This effect operated through early life stressors (e.g., parental divorce, school failure) and increased sensitivity to later neighborhood problems. Both of these mechanisms from early childhood neighborhood disadvantage to poor mental health could extend to women’s increased vulnerability to IPV (Yakubovich et al., 2018). Family-level stressors and buffers as well as mental health-related risks (e.g., alcohol/substance misuse, low self-efficacy) are likely on the causal pathway from early neighborhood deprivation exposure to early adulthood experiences of IPV—which is important to test in future research.

Persistent effects of childhood neighborhood disadvantage on later well-being are also supported in the (limited) experimental literature on neighborhood effects. In their reanalysis of Moving to Opportunity, Chetty and colleagues found that children under 13 whose families received a housing voucher to move to lower-poverty neighborhoods had higher incomes and were more likely to attend college and live in lower-poverty neighborhoods in early adulthood relative to those whose families received services as usual (Chetty et al., 2016). In contrast, housing vouchers did not improve later outcomes for children aged 13–18 at the start of the experiment: indeed, outcomes tended to worsen for children as a function of age. The researchers highlight the potential transition cost of moving neighborhoods, which, without active supportive interventions, may require earlier and longer exposure to the benefits of more structurally advantaged neighborhood environments to overcome (Bergman et al., 2019; Chetty et al., 2016). We may not have observed as strong contrasting results in the odds of IPV among participants who experienced increasing neighborhood deprivation exposure versus chronic high or decreasing exposure because these participants still spent a significant part of their childhoods in deprived neighborhoods.

The potential mechanisms underlying the relationship between early structural disadvantages and poor well-being outcomes, including IPV, likely involve social processes, such as social learning, norm-setting, and socialization (Herrenkohl et al., 2020; Voith, 2017; Voith et al., 2019). Neighborhood deprivation reduces the availability of resources in school, family, and work environments, which can in turn marginalize individuals from resource access or social mobility even when they transition to different neighborhoods (Sharkey & Elwert, 2011; Sharkey & Faber, 2014). These social and structural stressors occur within and shape normative contexts, including around gender and violence (e.g., the acceptability of violence; masculinity entailing power and dominance); the effects of early neighborhood deprivation on IPV may depend upon or interact with these social norms and processes of socialization. In conjunction with structural marginalization, these social processes could serve to explain why moving out of more disadvantaged neighborhoods may not be a panacea for those who have experienced early or sustained exposure. This requires further inquiry, including better understanding the role of gender in the effects of neighborhood deprivation on IPV victimization and perpetration (e.g., via gender role socialization).

## Strengths and Limitations

Our analyses are correlational in nature. We accounted for confounding by baseline family socioeconomic and psychosocial covariates. We did not account for later family characteristics as these are likely on the causal pathway, which would underestimate effects and potentially induce collider-stratification bias (Hernán et al., 2004; Wodtke et al., 2011). There may, however, be residual time-varying confounding affected by prior exposure. It is not possible to account for time-varying confounding without altering the latent class measurement model and the aim of the current study was to characterize IPV odds by within-participant change in neighborhood deprivation exposure over time. A prior study found consistent results in the association between long-term neighborhood deprivation exposure and IPV when time-varying confounding was and was not accounted for, which suggests that this is unlikely to explain away our findings (Yakubovich, Heron, Feder et al., 2020). Nonetheless, even without confirming causal hypotheses, our results suggest an important distribution of IPV risk by neighborhood exposure patterns that should be evaluated in future causal inference research and considered in intervention targets.

We dichotomized neighborhood deprivation exposure to create the most meaningful contrast between deprived and non-deprived neighborhoods with the available data, in line with prior neighborhood effects literature, which has often hypothesized threshold effects (Voith, 2017). However, the ALSPAC cohort is a higher socioeconomic sample living in less deprived neighborhoods compared to the national average (Boyd et al., 2013). Therefore, our results are likely conservative; this should be tested in future research with more diverse samples and across contexts, with attention paid to intersectional hypotheses (e.g., differences based on individual-level socioeconomic status, gender and sexual identities, and race). Only a small proportion of participants experienced a trajectory of increasing neighborhood deprivation exposure, further highlighting the importance of this replication—although our point and interval estimates of associations with IPV suggested meaningful differences from the chronic high and decreasing deprivation trajectory groups. Dichotomization was also analytically necessary to produce a parsimonious and theoretically meaningful classification of deprivation exposure patterns from the 2^10 (1,024) possible exposure patterns—as opposed to using the available quintiles (5^10 or 9,765,625 possible patterns). Testing robustness with alternative measures of neighborhood deprivation is an important future research direction.

We did not have data on women’s partners. An important direction for future research is to consider how the longitudinal exposure histories of each partner affect the risk of violence in the relationship. This should include considerations around mechanisms to different patterns of IPV victimization and perpetration and the potential interactive influences of factors such as gender role socialization.

Despite these limitations, the current study followed a cohort of participants from birth until early adulthood, who had substantial variability in longitudinal neighborhood deprivation exposures and IPV to characterize these associations. We used validated measures, accounted for a rich set of socioeconomic and psychosocial covariates measured at baseline (to avoid over-adjustment) and misclassification bias, and demonstrated robustness in our estimation to including more or less missing data.

## Conclusion

Women who lived in deprived neighborhoods during their early childhoods, regardless of their later neighborhood environments, had higher odds of experiencing IPV in early adulthood than those who consistently lived in non-deprived neighborhoods. Our results demonstrate the importance of moving beyond considering only mechanisms related to current neighborhood environments (e.g., via social disorganization and collective efficacy theories) to investigate possible developmental pathways. Interventions that target the structural determinants of neighborhood deprivation and social (im)mobility across neighborhoods may reduce IPV against women. Our results suggest that structural interventions targeting neighborhood deprivation (on their own) may be most effective at reducing later IPV risk among those who experience these changes in early childhood. Future research is needed to test underlying mechanisms and generalizability to other contexts.

## Supplemental Material 

Supplemental material for Trajectories of Exposure to Neighborhood Deprivation and the Odds of Experiencing Intimate Partner Violence Among Women: Are There Sensitive Periods for Exposure? Click here for additional data file. Supplemental material for Trajectories of Exposure to Neighborhood Deprivation and the Odds of Experiencing Intimate Partner Violence Among Women: Are There Sensitive Periods for Exposure? by Alexa R. Yakubovich, Jon Heron, Christine Barter, and David K. Humphreys in Journal of Interpersonal Violence
**Figure A1.** Trajectories of Neighborhood Deprivation for Participants with at Least 50% Non-Missing Time Points (N=4,058).
**Table A1.** Association of Neighborhood Deprivation Trajectory Group Membership with IPV at Age 21 Years (N=2,815).
**Table A2.** Association of Neighborhood Deprivation Trajectory Group Membership with IPV at Age 21 Years (N=1,456).
**Table A3:** Association of Neighborhood Deprivation Trajectory Group Membership with IPV at Age 21 Years (N=1,353).

## Author Biographies

**Alexa R. Yakubovich**, PhD, is a CIHR Health System Impact and CIHR-CMHC Postdoctoral Fellow at the Centre for Urban Health Solutions at Unity Health Toronto and the University of Toronto. She received her PhD in Evidence-based Social Intervention and Policy Evaluation from the University of Oxford. Alexa’s research focuses on the social and structural determinants of interpersonal violence and related health inequities and the design and evaluation of preventive interventions.

**Jon Heron**, PhD, is a senior research fellow in the School of Social and Community Medicine at the University of Bristol. He is a statistician with over 15 years of experience working with the Avon Longitudinal Study of Parents and Children. His methodological interests include longitudinal modeling and latent variable analysis.

**Christine Barter**, PhD, is a reader in Young People and Violence Prevention in the Connect Centre for International Research on New Approaches to Prevent Violence and Harm at the University of Lancashire. Her research has predominantly focused on children’s and young people’s experiences of abuse and violence. Her recent work has addressed prevention of violence and abuse in young people’s relationships.

**David K. Humphreys**, PhD, is an associate professor of Evidence-based Intervention and Policy Evaluation in the Department of Social Policy and Intervention at the University of Oxford. He is an interdisciplinary social scientist whose research spans criminology, social (or public) policy, public health, and epidemiology. His main topic of interest is the causes, consequences, and prevention of violence and injury.

## References

[bibr1-0886260520959626] Barter C., McCarry M., Berridge D., & Evans K. (2009). *Partner exploitation and violence in teenage intimate relationships* . NSPCC/University of Bristol.

[bibr2-0886260520959626] Barter C., Stanley N., Wood M., Lanau A., Aghtaie N., & Larkins C. (2017). Young people’s online and face-to-face experiences of interpersonal violence and abuse and their subjective impact across five European countries. *Psychology of Violence* , 7(3), 375–384.

[bibr3-0886260520959626] Benson M. L., Fox G. L., DeMaris A., & Wyk J. A. van. (2003). Neighborhood disadvantage, individual economic distress and violence against women in intimate relationships. *Journal of Quantitative Criminology* , 19, 207–235. http://ovidsp.ovid.com/ovidweb.cgi?T=JS&PAGE=reference&D=psyc4&NEWS=N&AN=2003-99726-001

[bibr4-0886260520959626] Bergman P., Chetty R., DeLuca S., Hendren N., Katz L. F., & Palmer C. (2019). *Creating moves to opportunity: Experimental evidence on barriers to neighborhood choice* (NBER Working Paper No. 26164). https://scholar.harvard.edu/lkatz/publications/creating-moves-opportunity-experimental-evidence-barriers-neighborhood-choice

[bibr5-0886260520959626] Beyer K., Wallis A. B., & Hamberger L. K. (2015). Neighborhood environment and intimate partner violence: A systematic review. *Trauma, Violence, & Abuse* , 16(1), 16–47. 10.1177/1524838013515758 PMC4476540 24370630

[bibr6-0886260520959626] Boyd A., Golding J., Macleod J., Lawlor D. A., Fraser A., Henderson J., Molloy L., Ness A., Ring S., & Davey Smith G. (2013). Cohort profile: The “children of the 90s”—The index offspring of the Avon Longitudinal Study of Parents and Children. *International Journal of Epidemiology* , 42(1), 111–127. 10.1093/ije/dys064 22507743 PMC3600618

[bibr7-0886260520959626] Bristol City Council. (2011). *Deprivation in Bristol 2010: The mapping of deprivation within Bristol Local Authority Area* . Author.

[bibr8-0886260520959626] Browning C. R. (2002). The span of collective efficacy: Extending social disorganization theory to partner violence *Journal of Marriage and Family* , 64(4), 833–850.

[bibr9-0886260520959626] Campbell J. C. (2002). Health consequences of intimate partner violence. *The Lancet* , 359(9314), 1331–1336. 10.1016/s0140-6736(02)08336-8 11965295

[bibr10-0886260520959626] Capaldi D. M., Knoble N. B., Shortt J. W., & Kim H. K. (2012). A systematic review of risk factors for intimate partner violence. *Partner Abuse* , 3(2), 231–280. 10.1891/1946-6560.3.2.231 22754606 PMC3384540

[bibr11-0886260520959626] Cerda M., Tracy M., Ahern J., & Galea S. (2014). Addressing population health and health inequalities: The role of fundamental causes. *American Journal of Public Health* , 104(S4), S609-S619. 10.2105/AJPH.2014.302055) 25100428 PMC4126171

[bibr12-0886260520959626] Cerda M., Tracy M., Keyes K. M., & Galea S. (2015). To treat or to prevent? Reducing the population burden of violence-related post-traumatic stress disorder. *Epidemiology* , 26(5), 681–689. 10.1097/EDE.0000000000000350 26237744 PMC4827920

[bibr13-0886260520959626] Chetty R., Hendren N., & Katz L. F. (2016). *The effects of exposure to better neighbourhoods on children: New evidence from the moving to opportunity experiment* (NBER Working Paper No. 21156). https://scholar.harvard.edu/files/lkatz/files/chk_aer_mto_0416.pdf 10.1257/aer.20150572 29546974

[bibr14-0886260520959626] Cox J. L., Holden J. M., & Sagovsky R. (1987). Detection of postnatal depression: Development of the 10-item Edinburgh Postnatal Depression Scale. *British Journal of Psychiatry* , 150, 782–786. 10.1192/bjp.150.6.782 3651732

[bibr15-0886260520959626] DeMaris A., Benson M. L., Fox G. L., Hill T. D., & Wyk J. V. (2003). Distal and proximal factors in domestic violence: A test of an integrated model. *Journal of Marriage and Family* , 65, 652–667.

[bibr16-0886260520959626] Fox G. L., Benson M. L., DeMaris A. A., & Wyk J. van. (2002). Economic distress and intimate violence: Testing family stress and resources theories. *Journal of Marriage and the Family, Living, Marriage and Family Living* , 64, 793–807. http://ovidsp.ovid.com/ovidweb.cgi?T=JS&PAGE=reference&D=psyc4&NEWS=N&AN=2002-17716-017

[bibr17-0886260520959626] Fraser A., Macdonald-Wallis C., Tilling K., Boyd A., Golding J., Davey Smith G., Henderson J., Macleod J., Molloy L., Ness A., Ring S., Nelson S. M., & Lawlor D. A. (2013). Cohort profile: The Avon Longitudinal Study of Parents and Children—ALSPAC mothers cohort. *International Journal of Epidemiology* , 42(1), 97–110. 10.1093/ije/dys066 22507742 PMC3600619

[bibr18-0886260520959626] Garcia-Moreno C., Pallitto C., Devries K., Stockl H., Watts C., & Abrahams N. (Eds.). (2013). *Global and regional estimates of violence against women: Prevalence and health effects of intimate partner violence and non-partner sexual violence* . World Health Organization.

[bibr19-0886260520959626] Giordano P. C., Copp J. E., Longmore M. A., & Manning W. D. (2016). Anger, control, and intimate partner violence in young adulthood. *Journal of Family Violence* , 31(1), 1–13. 10.1007/s10896-015-9753-3 26924886 PMC4767526

[bibr20-0886260520959626] Gomez A. M. (2011). Testing the cycle of violence hypothesis: Child abuse and adolescent dating violence as predictors of intimate partner violence in young adulthood. *Youth & Society* , 43(1), 171–192. 10.1177/0044118X09358313

[bibr21-0886260520959626] Hernán M. A., Hernández-Díaz S., & Robins J. M. (2004). A structural approach to selection bias. *Epidemiology* , 15(5), 615–625. 10.1097/01.ede.0000135174.63482.43 15308962

[bibr22-0886260520959626] Heron J., Croudace T., Barker E., & Tilling K. (2015). A comparison of approaches for assessing covariate effects in latent class analysis. *Longitudinal and Life Course Studies* , 6(4). 10.14301/llcs.v6i4.322

[bibr23-0886260520959626] Herrenkohl T. I., Fedina L., Roberto K. A., Raquet K. L., Hu R. X., Rousson A. N., & Mason W. A. (2020). Child Maltreatment, Youth Violence, Intimate Partner Violence, and Elder Mistreatment: A Review and Theoretical Analysis of Research on Violence Across the Life Course. *Trauma Violence Abuse* . Advance online publication. 10.1136/bmjopen-2014-007141 PMC10202370 32723166

[bibr23a-0886260520959626] Hester M., Ferrari G., Jones S. K., Williamson E., Bacchus L. J., Peters T. J., & Feder G. (2015). Occurrence and impact of negative behaviour, including domestic violence and abuse, in men attending UK primary care health clinics: A cross-sectional survey. *BMJ Open* , 5(5), e007141. 10.1136/bmjopen-2014-007141 PMC4452742 25991450

[bibr24-0886260520959626] Jain S., Buka Stephen L., Subramanian S. V., & Molnar Beth E. (2010). Neighborhood predictors of dating violence victimization and perpetration in young adulthood: A multilevel study. *American Journal of Public Health* , 100(9), 1737–1744. https://www.ncbi.nlm.nih.gov/pmc/articles/PMC2920975/pdf/1737.pdf 20634470 10.2105/AJPH.2009.169730 PMC2920975

[bibr25-0886260520959626] Jivraj S., Murray E. T., Norman P., & Nicholas O. (2019). The impact of life course exposures to neighbourhood deprivation on health and well-being: A review of the long-term neighbourhood effects literature. *European Journal of Public Health* . 10.1093/eurpub/ckz153 PMC8489013 31576400

[bibr26-0886260520959626] Leddy A. M., Lippman S. A., Neilands T. B., Twine R., Ahern J., Gomez-Olive F. X., DeLong S. M., MacPhail C., Kahn K., & Pettifor A. E. (2018). Community collective efficacy is associated with reduced physical intimate partner violence (IPV) incidence in the rural province of Mpumalanga, South Africa: Findings from HPTN 068. *Journal of Epidemiology & Community Health* . 10.1136/jech-2018-211357 PMC6490171 30455373

[bibr27-0886260520959626] McLennan D., Barnes H., Noble M., Davies J., & Garratt E. (2011). *The English Indices of Deprivation 2010* . Department for Communities and Local Government.

[bibr28-0886260520959626] Morris T., Manley D., & Ham M. van. (2018). Context or composition: How does neighbourhood deprivation impact upon adolescent smoking behaviour? *PLoS ONE* , 13(2), e0192566. 10.1371/journal.pone.0192566 29420655 PMC5805312

[bibr29-0886260520959626] Muthén L. K., & Muthén B. O. (2017). *Mplus: User’s guide (8th ed.)* . Muthén & Muthén.

[bibr30-0886260520959626] Nagin D. S. (2005). *Group-based modeling of development* . Harvard University Press.

[bibr31-0886260520959626] Sampson R. J., Morenoff J. D., & Gannon-Rowley T. (2002). Assessing “neighborhood effects”: Social processes and new directions in research. *Annual Review of Sociology* , 28(1), 443–478. 10.1146/annurev.soc.28.110601.141114

[bibr32-0886260520959626] Sampson R. J., Raudenbush S. W., & Earls F. (1997). Neighbourhoods and violent crime: A multilevel study of collective efficacy. *Science* , 277, 918–924. 9252316 10.1126/science.277.5328.918

[bibr33-0886260520959626] Sharkey P., & Elwert F. (2011). The legacy of disadvantage: Multigenerational neighborhood effects on cognitive ability. *American Journal of Sociology* , 116(6), 1934–1981. 10.1086/660009 PMC3286027 21932471

[bibr34-0886260520959626] Sharkey P., & Faber J. W. (2014). Where, When, why, and for whom do residential contexts matter? Moving away from the dichotomous understanding of neighborhood effects. *Annual Review of Sociology* , 40(1), 559–579. 10.1146/annurev-soc-071913-043350

[bibr35-0886260520959626] Shaw C. R., & McKay H. D. (1942). *Juvenile delinquency and urban areas: A study of rates of delinquents in relation to differential characteristics of local communities in American cities* . University of Chicago Press.

[bibr36-0886260520959626] Smith-Marek E. N., Cafferky B., Dharnidharka P., Mallory A. B., Dominguez M., High J., Stith S. M., & Mendez M. (2015). Effects of childhood experiences of family violence on adult partner violence: A meta-analytic review. *Journal of Family Theory & Review* , 7(4), 498–519. 10.1111/jftr.12113

[bibr37-0886260520959626] Thulin E. J., Heinze J. E., Kusunoki Y., Hsieh H-F., & Zimmerman M. A. (2020). Perceived neighborhood characteristics and experiences of intimate partner violence: A multilevel analysis. *Journal of Interpersonal Violence* , 1–23. 10.1177/0886260520906183 PMC12211242 32054385

[bibr38-0886260520959626] Vanderende K. E., Yount K. M., Dynes M. M., & Sibley L. M. (2012). Community-level correlates of intimate partner violence against women globally: A systematic review. *Social Science & Medicine* , 75(7), 1143–1155. 10.1016/j.socscimed.2012.05.027 22762950

[bibr39-0886260520959626] Vermunt J. K. (2010). Latent class modeling with covariates: Two improved three-step approaches. *Political Analysis* , 18(4), 450–469. 10.1093/pan/mpq025

[bibr40-0886260520959626] Voith L. A. (2017). Understanding the relation between neighborhoods and intimate partner violence: An integrative review. *Trauma, Violence, & Abuse* , 1–13. 10.1177/1524838017717744 29333974

[bibr41-0886260520959626] Voith L. A., Topitzes J., & Berg K. A. (2019). The transmission of violence and trauma across development and environmental contexts: Intimate partner violence from the perspective of men with histories of perpetration. *Child Abuse & Neglect* , 99, 104267. 10.1016/j.chiabu.2019.104267 31743807

[bibr42-0886260520959626] Wheaton B., & Clarke P. (2003). Space meets time: Integrating temporal and contextual influences on mental health in early adulthood. *American Sociological Review* , 68(5), 680–706.

[bibr43-0886260520959626] Wodtke G. T. (2013). Duration and timing of exposure to neighborhood poverty and the risk of adolescent parenthood. *Demography* , 50, 1765–1788. 10.1007/s13524-013-0219-z) 23720166 PMC3882124

[bibr44-0886260520959626] Wodtke G. T., Harding D. J., & Elwert F. (2011). Neighborhood effects in temporal perspective. *American Sociological Review* , 76(5), 713–736. 10.1177/0003122411420816 22879678 PMC3413291

[bibr45-0886260520959626] van Wyk J. A., Benson M. L., Fox G. L., & DeMaris A. (2003). Detangling individual-, partner-, and community-level correlates of partner violence. *Crime & Delinquency* , 49(3), 412–438. 10.1177/0011128703253763

[bibr46-0886260520959626] Yakubovich A. R., Heron J., Feder G., Fraser A., & Humphreys D. K. (2019). Intimate partner violence victimisation in early adulthood: Psychometric properties of a new measure and gender differences in the Avon Longitudinal Study of Parents and Children. *BMJ Open* , 9(3), e025621. 10.1136/bmjopen-2018-025621 PMC6475136 30904864

[bibr47-0886260520959626] Yakubovich A. R., Heron J., Feder G., Fraser A., & Humphreys D. K. (2020). Long-term exposure to neighborhood deprivation and intimate partner violence among women: A UK birth-cohort study. *Epidemiology* , 31(2), 272–281. 10.1097/EDE.0000000000001144 31764275 PMC7004477

[bibr48-0886260520959626] Yakubovich A. R., Heron J., & Humphreys D. K. (2020). How do perceived and objective measures of neighbourhood disadvantage vary over time? Results from a prospective-longitudinal study in the UK with implications for longitudinal research on neighbourhood effects on health. *PLoS ONE* . 10.1371/journal10.1371/journal.pone.0231779 PMC7162465 32298364

[bibr49-0886260520959626] Yakubovich A. R., Stöckl H., Murray J., Melendez-Torres G. J., Steinert J. I., Glavin C. E. Y., & Humphreys D. K. (2018). Risk and protective factors for intimate partner violence against women: Systematic review and meta-analyses of prospective–longitudinal studies. *American Journal of Public Health* , 108(7), e1–e11. 10.2105/ajph.2018.304428 PMC5993370 29771615

