# Supplementary material for: Trajectories of Exposure to Neighborhood Deprivation and the Odds of Experiencing Intimate Partner Violence Among Women: Are There Sensitive Periods for Exposure?
Source: J Interpers Violence. 2020 Sep 22;37(7-8):NP5728–46. doi: 10.1177/0886260520959626 (PMC8980452; doi:10.1177/0886260520959626)
Supplement: Supplemental material for Trajectories of Exposure to Neighborhood Deprivation and the Odds of Experiencing Intimate Partner Violence Among Women: Are There Sensitive Periods for Exposure? [file 959626_Suppl._Material.pdf]

Appendix A. Sensitivity Analyses.

Figure A1. Trajectories of Neighborhood Deprivation for Participants with at Least 50% Non-Missing Time Points (N=4,058).

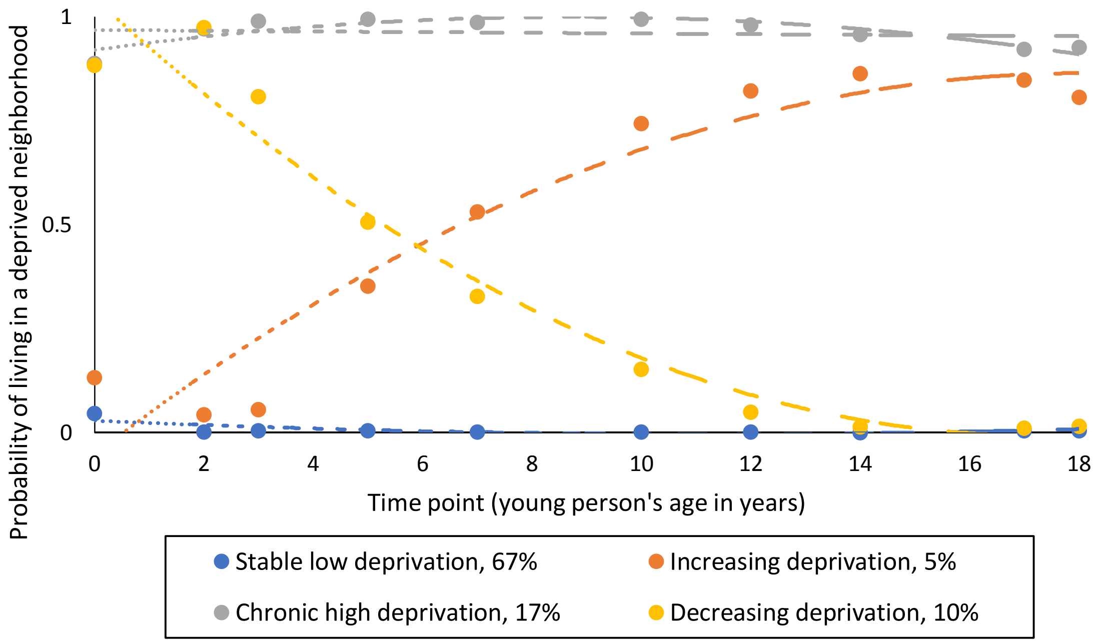

Note. Proportions for each trajectory group are based on the estimated model; dots represent observed means and dotted lines represent estimated trajectories. Entropy=0.96.

Distal Outcome Models with Increasingly Strict Missing Data Exclusion Criteria

Table A1. Association of Neighborhood Deprivation Trajectory Group Membership with IPV at Age 21 Years (N=2,815).

Trajectory group	Odds ratio	95% CI
Stable low deprivation	Referent	
Increasing deprivation	1.32	0.73-2.37
Chronic high deprivation	1.54	1.06-2.25
Decreasing deprivation	1.55	1.03-2.33

Note. N=2,815 participants with at least 50% of neighborhood deprivation data over the study period (i.e., at least 5 out of 10 time points) and all baseline covariates. Entropy=0.93.

Table A2. Association of Neighborhood Deprivation Trajectory Group Membership with IPV at Age 21 Years (N=1,456).

**Trajectory group**	**Odds ratio**	**95% CI**
Stable low deprivation	Referent	
Increasing deprivation	1.32	0.74-2.36
Chronic high deprivation	1.53	1.02-2.31
Decreasing deprivation	1.70	1.03-2.83

Note. N=1,456 participants with at least 1 time point of neighborhood deprivation data over the study period, IPV data at age 21, and all baseline covariates. Entropy=0.80.

Table A3: Association of Neighborhood Deprivation Trajectory Group Membership with IPV at Age 21 Years (N=1,353).

**Trajectory group**	**Odds ratio**	**95% CI**
Stable low deprivation	Referent	
Increasing deprivation	1.32	0.73-2.38
Chronic high deprivation	1.54	1.06-2.25
Decreasing deprivation	1.55	1.03-2.33

Note. N=1,353 participants with at least 50% of neighborhood deprivation data over the study period (i.e., at least 5 out of 10 time points), IPV data at age 21, and all baseline covariates. Entropy=0.94.
